# Hypoxia stimulates the expression of macrophage migration inhibitory factor in human vascular smooth muscle cells via HIF-1α dependent pathway

**DOI:** 10.1186/1471-2121-11-66

**Published:** 2010-08-20

**Authors:** Hua Fu, Fengming Luo, Li Yang, Wenchao Wu, Xiaojing Liu

**Affiliations:** 1 Department of Cardiology, West China Hospital, Sichuan University, Chengdu 610041, China; 2 Laboratory of Cardiovascular Diseases, Regenerative Medicine Research Center, West China Hospital, Sichuan University, Chengdu 610041, China; 3 Golden-card ward, West China Hospital, Sichuan University, Chengdu 610041, China; 4 Department of Digestive Diseases, West China Hospital, Sichuan University, Chengdu 610041, China

## Abstract

**Background:**

Hypoxia plays an important role in vascular remodeling and directly affects vascular smooth muscle cells (VSMC) functions. Macrophage migration inhibitory factor (MIF) is a well known proinflammatory factor, and recent evidence suggests an important role of MIF in the progression of atherosclerosis and restenosis. However, the potential link between hypoxia and MIF in VSMC has not been investigated. The current study was designed to test whether hypoxia could regulate MIF expression in human VSMC. The effect of modulating MIF expression on hypoxia-induced VSMC proliferation and migration was also investigated at the same time.

**Results:**

Expression of MIF mRNA and protein was up-regulated as early as 2 hours in cultured human VSMCs after exposed to moderate hypoxia condition (3% O_2_). The up-regulation of MIF expression appears to be dependent on hypoxia-inducible transcription factor-1α(HIF-1α) since knockdown of HIF-1α inhibits the hypoxia induction of MIF gene and protein expression. The hypoxia induced expression of MIF was attenuated by antioxidant treatment as well as by inhibition of extracellular signal-regulated kinase (ERK). Under moderate hypoxia conditions (3% O_2_), both cell proliferation and cell migration were increased in VSMC cells. Blocking the MIF by specific small interference RNA to MIF (MIF-shRNA) resulted in the suppression of proliferation and migration of VSMCs.

**Conclusion:**

Our results demonstrated that in VSMCs, hypoxia increased MIF gene expression and protein production. The hypoxia-induced HIF-1α activation, reactive oxygen species (ROS) generation and ERK activation might be involved in this response. Both MIF and HIF-1α mediated the hypoxia response of vascular smooth muscle cells, including cell migration and proliferation.

## Background

Tissue hypoxia is an essential feature of chronic inflammatory diseases. In the cardiovascular system, for example, when arterial wall thickens and blood-diffusion capacity is low in atherosclerotic lesions, hypoxia plays a key role in the development of atherosclerosis [1,2]. The cellular effects of hypoxia are primarily mediated by the hypoxia-inducible transcription factor-1 (HIF-1). It is a heterodimeric transcription factor composed of α and β subunits. HIF-1β is constitutively expressed in many cell types. HIF-1α, the active subunit of HIF-1, is undetectable under normoxia because of rapid proteasomal degradation. But it is stabilized under hypoxia conditions [3]. HIF-1 specifically binds hypoxic response element (HRE)-driven promoters on a number of genes such as vascular endothelial growth factor (VEGF), heme oxygenase and erythropoietin. In human atherosclerosis, HIF-1α protein co-localizes with macrophages [2]. HIF-1α may play a role in foam cell formation [4]. Evidences suggest that the HIF-1 pathway is associated with the progression and angiogenesis of human atherosclerosis [2,5,6]. Recent studies have shown that in normal oxygen conditions, G-protein-coupled receptor agonists including angiotensin II [7,8] and thrombin [9], potently induce and activate HIF-1α in vascular smooth muscle cells. These results suggest a more general role of this transcription factor in the vascular response to injury. However, the role of hypoxia and HIF-1 in atherosclerosis remains largely unknown.

Recently, macrophage migration inhibitory factor (MIF) has emerged as a key factor in vascular remodeling and in the development and progression of atherosclerosis [10-13]. MIF is an essential, upstream component of the inflammatory cascade and has a critical role in several inflammatory conditions [10]. It can be expressed by vascular endothelial cells, VSMCs and macrophages. Increased expression of vascular MIF is associated with foam cell transformation during atherogenesis. MIF is expressed in atherosclerotic lesions, and has been suggested to be involved in atherosclerotic plaque development [12]. Several pro-atherosclerotic mediators such as oxidized LDL [14], CD40-L and angiotensin II are able to stimulate MIF expression [12]. However, the regulation of MIF expression in vascular cells, and its mechanisms of action have received little attention in atherosclerosis research.

MIF has recently been shown to be up-regulated by hypoxia in several tumor cell types in vitro including breast carcinoma cells [15,16]. However, there are few data about the direct effects of hypoxia on the expression of MIF in VSMCs. VSMCs are one of the major constituents of blood vessels. VSMCs are also essential to atherosclerotic lesions. In the view of the increased expression of MIF in the atherosclerosis, we hypothesized that MIF might be up-regulated by hypoxia in VSMCs, and the up-regulation of MIF could be mediated via HIF-1 dependent pathway. In order to test our hypothesis, we examined the influence of hypoxia on the MIF expression in human VSMCs. Primary human umbilical artery smooth muscle cells (HUASMCs) were used as a model to study the effects of hypoxia on the expression of MIF modulated by RNA interference in this study.

## Results

### Hypoxia increases MIF expression in cultured vascular smooth muscle cells

It was reported that hypoxia (1% O_2_) stimulation could induce MIF expression in breast cancer cell line [16]. However, exposure to hypoxia (1% O_2_) reduced cell proliferation and increased apoptotic cell death in VSMC [17]. Therefore, in our present study, in order to test the effect of hypoxia on the MIF expression, different degrees of hypoxia (10, 5, or 3% O_2_, but not 1% O_2_) were used.

**Figure 1:**
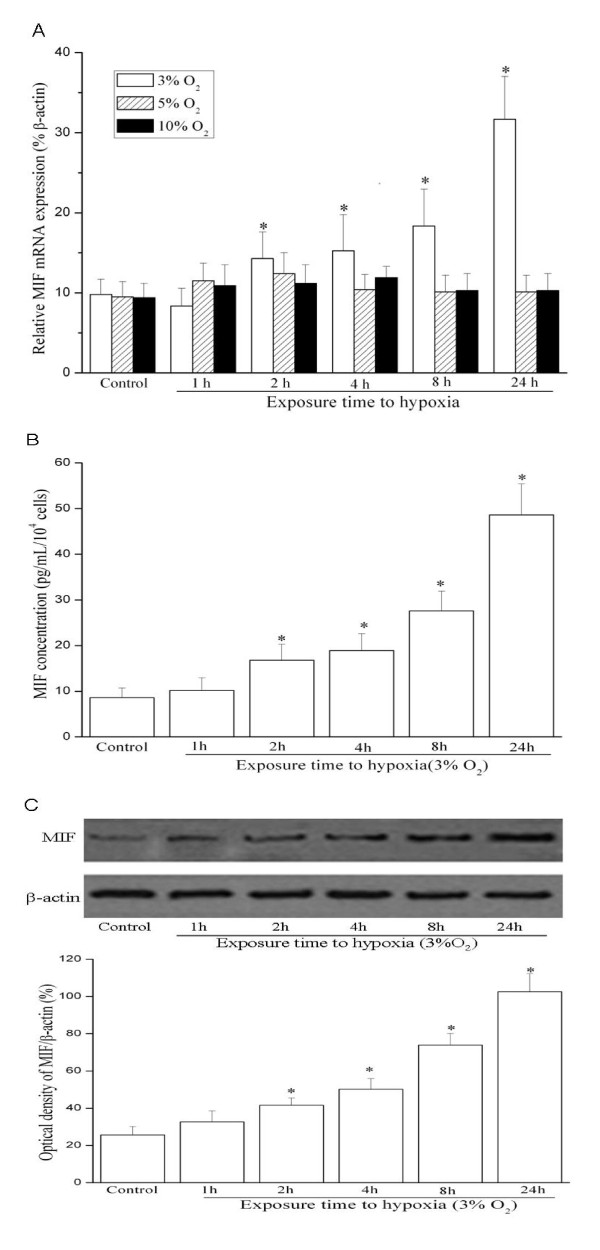
**Hypoxia increases MIF mRNA expression (A) and protein production (B, C) in HUASMCs**. Growth-arrested HUASMCs were exposed to hypoxia for different durations. (A) Quantitative RT-PCR (Q-PCR) results. Total cellular RNA was isolated from normoxia or hypoxia-stimulated cells. After reverse transcription, they were subjected to quantitative PCR analysis to determine MIF mRNA level. Graph is representative of relative MIF mRNA levels in the various conditions (n = 3 in each group). * indicates *P *< 0.05 *vs *control cells under normoxia. (B) Release of MIF protein measured by ELISA. Growth-arrested cells were exposed to hypoxia (3% O_2_) for different durations, and MIF protein released into cell culture media was measured by ELISA (n = 3 in each group). * indicates *P *< 0.05 *vs *control cells under normoxia. (C) Expression of MIF protein detected by Western blot. Western Blot detecting of MIF expression in control HUASMCs under normoxia or hypoxia (3% O_2_) for different durations. Representative Western blot (top) and values of MIF production (mean ± SEM of 3 experiments, bottom). Results of MIF protein production were obtained from densitometric analysis and expressed as ratio of MIF/β-actin. * *P *< 0.05 *vs *control cells under normoxia.

MIF mRNA expression was evaluated by quantitative RT-PCR in HUASMCs exposed to different oxygen level for different time. As shown in Figure 1A, hypoxia at 3% oxygen for 2 h significantly induced MIF mRNA expression, whereas hypoxia at 5 and 10% oxygen had no effect on MIF gene expression. The induction was peaked at 24 h after exposure to hypoxia (3% O_2_). We then used 3% oxygen treatment for 24 h as hypoxia stimulation in the following experiments.

We next examined whether MIF protein production was regulated in response to hypoxia by Western Blot and ELISA. The cells were exposed to hypoxia for indicated times. Subsequently, the secretion of MIF in the culture media was measured by ELISA. Under normoxia condition, growth-arrested HUASMCs expressed low level of MIF protein. Our data showed that 3% oxygen induced maximum MIF protein expression at 24 h (Figure 1B). Similarly, under normoxia condition, HUASMCs only expressed low level MIF protein as detected by Western blot. Total cellular MIF protein levels began to increase 2 h after exposure to 3% O_2_, and peaked at 24 h (Figure 1C). These data suggest that hypoxia induces both MIF mRNA and protein production in HUASMCs.

### Hypoxia stimulation (3% O_2_) induces expression and activation of HIF-1α in cultured vascular smooth muscle cells

**Figure 2:**
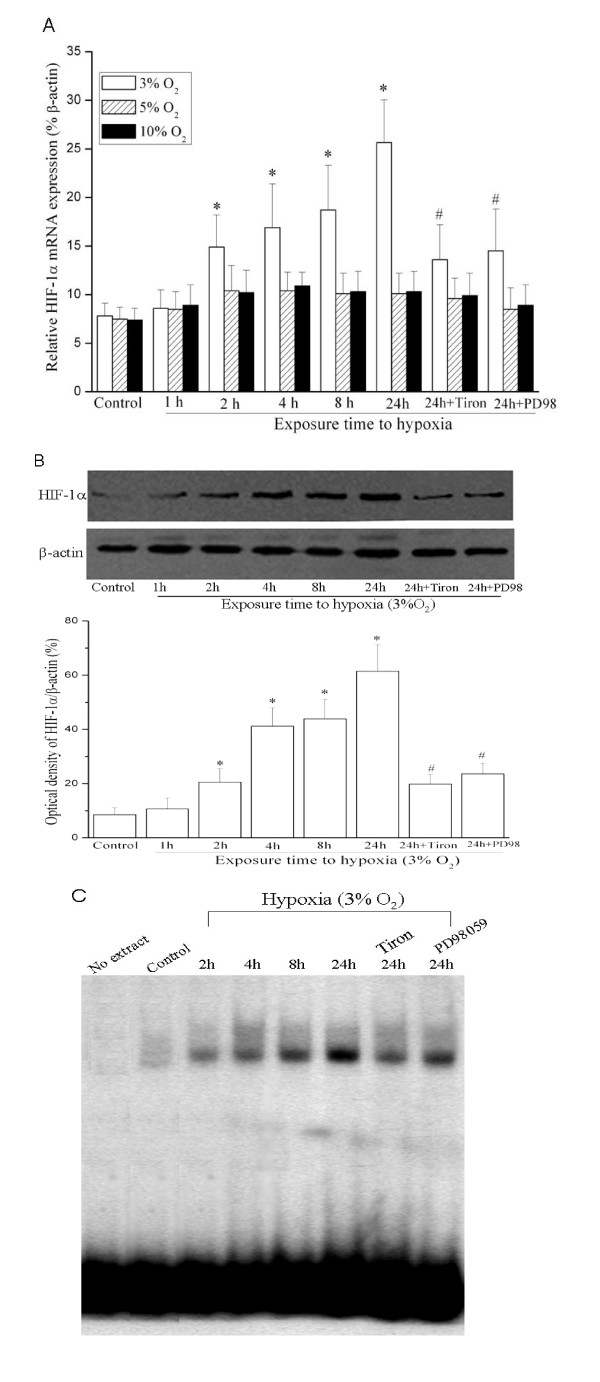
**Hypoxia stimulation (3% O_2_) induces expression and activation of HIF-1α in cultured HUASMCs**. (A) Hypoxia (3% O_2_) induces HIF-1α mRNA expression. Total cellular RNA was isolated from control HUASMCs under normoxia or hypoxia-stimulated cells in the presence or absence of inhibitors. After reverse transcription, they were subjected to quantitative PCR analysis to determine HIF-1α mRNA level. Graph is representative of relative HIF-1α mRNA levels in the various conditions (n = 3 in each group). * indicates *P *< 0.05 *vs *control cells under normoxia. # *P *< 0.05 *vs *cells exposed to hypoxia for 24 h. *PD98: PD98059*. (B) Hypoxia (3% O_2_) increases HIF-1α protein levels. Western Blot detecting of HIF-1α protein expression in control HUASMCs under normoxia or cells exposed to hypoxia (3% O_2_) in the presence or absence of inhibitors. Representative Western blot (top) and values of HIF-1α production (mean ± SEM of 3 experiments, bottom). Results of HIF-1α protein production were obtained from densitometric analysis and expressed as ratio of HIF-1α/β-actin. * *P *< 0.05 *vs *control cells under normoxia. # *P *< 0.05 *vs *cells exposed to hypoxia for 24 h. *PD98: PD98059*. (C) Hypoxia increases HIF-1α DNA binding activity (EMSA results). Representative electrophoretic mobility shift assay (EMSA) showing protein binding to the HIF-1α oligonucleotide in nuclear extracts of HUASMCs after hypoxia stimulation in the presence or absence of inhibitors. Similar results were found in another two independent experiments.

HIF-1α is the key master regulator to hypoxic response [3], so we observed the possible role of HIF-1α in our experimental model. HIF-1α mRNA and protein levels were highly induced in HUASMCs exposed to hypoxia (3% O_2_) for 24 h, coincident with the expression of MIF (Figure 2A and 2B).

In order to further validate that HIF-1α could be activated by the hypoxia stimulation (3% O_2_), we next performed electrophoretic mobility shift assay (EMSA) experiments aimed at assessing whether HIF-1α DNA binding activity can be induced under 3% O_2 _hypoxia conditions. EMSA assay was performed using primers encompassing the hypoxia response element (HRE) and adjacent flanking regions in the promoter of human MIF gene [18]. Our data showed that HIF-1α binding activity could be increased by exposure to hypoxia (3% O_2_) for 24 h (Figure 2C).

### The hypoxia-induced MIF expression in cultured vascular smooth muscle cells is dependent on HIF-1α pathway

**Figure 3:**
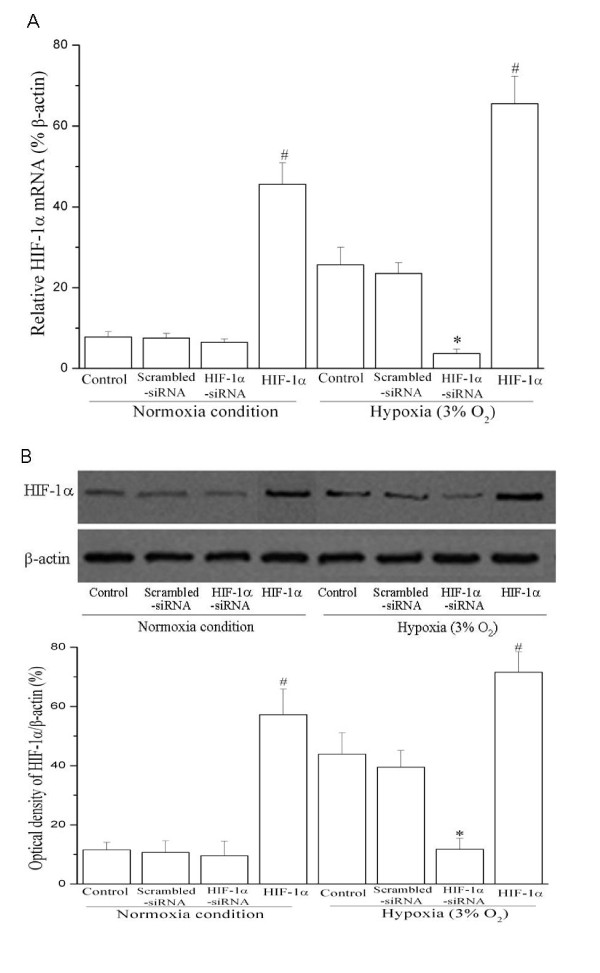
**Knockdown of HIF-1α mRNA and protein expression by RNA interference in HUASMCs**. Growth-arrested HUASMCs were transfected with wild type HIF-1α, HIF-1α-siRNA or scrambled siRNA expressing plasmids for 24 h and then exposed to normoxia or hypoxia (3% O_2_) for 24 h. (A) Inhibition HIF-1α mRNA expression in HUASMCs by siRNA. HIF-1α mRNA expression were assayed by Q-PCR (n = 3 in each group). *P < 0.05 *vs *control or scrambled siRNA transfection under hypoxia. # *P *< 0.05 *vs *control cells under normoxia or hypoxia conditions. (B)Suppression and over-expression of HIF-1α: western blot analysis. Representative Western blot (top) and values of HIF-1α production (mean ± SEM of 3 experiments, bottom). Results of HIF-1α protein production were obtained from densitometric analysis and expressed as ratio of HIF-1α/β-actin. *P < 0.05 *vs *control or scrambled siRNA transfection under hypoxia. # *P *< 0.05 *vs *control cells under normoxia or hypoxia conditions. *HIF-1: wild type HIF-1α plasmid transfection; Scrambled-siRNA: scrambled-siRNA plasmid transfection; HIF-1α-siRNA: HIF-1α-siRNA plasmid transfection*.

Next, we investigated whether HIF-1α was involved in hypoxia-induced MIF upregulation in HUASMCs. To block HIF-1α actions, we used a HIF-1α-specific small inhibitory RNA construct (HIF-1α-siRNA) to knock-down HIF-1α expression. The specific HIF-1α-siRNA expressing plasmid was constructed and used to knock-down HIF-1α expression in the lung cancer cell line A549 cells in our previous report [19]. In order to confirm the inhibition effect of HIF-1α-siRNA expressing plasmid, we determined the gene and protein expression of HIF-1α in HUASMCs after transfection. As shown in Figure 3A, the hypoxia-induced expression of HIF-1α mRNA was significantly suppressed in HUASMCs transfected with HIF-1α-siRNA vector. Accordingly, HIF-1α protein level was decreased in cells receiving HIF-1α-siRNA (Figure 3B). Transfection of HUASMCs with a wild-type HIF-1α expression vector led to up-regulation HIF-1α at both the mRNA and protein level under normoxic and hypoxic conditions (Figure 3).

**Figure 4:**
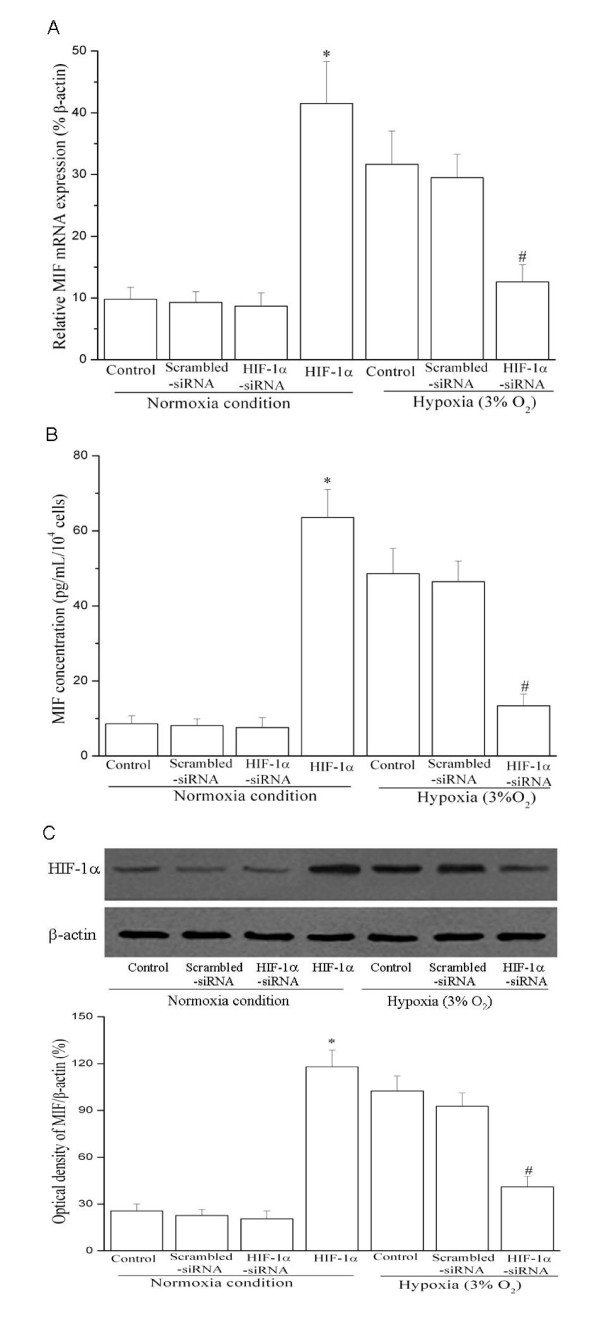
**Hypoxia induces MIF mRNA (A) and protein levels (B, C) via HIF-1α pathway in HUASMCs**. Growth-arrested HUASMCs were transfected with wild type HIF-1α, HIF-1α-siRNA or scrambled siRNA expressing plasmids for 24 h and then exposed to normoxia or hypoxia (3% O_2_) for 24 h. (A) Real-time PCR results. MIF mRNA expression were assayed by Q-PCR (n = 3 in each group). * *P *< 0.05 *vs *control cells under normoxia. # *P *< 0.05 *vs *control or scrambled siRNA transfection under hypoxia. (B) ELISA results. MIF protein released into cell culture media was measured by ELISA (n = 3 in each group). * indicates *P *< 0.05 *vs *control cells under normoxia. # *P *< 0.05 *vs *control or scrambled siRNA transfection under hypoxia. (C) Western blot results. Representative Western blot (top) and values of total MIF production (mean ± SEM of 3 experiments, bottom). Results of total MIF protein production were obtained from densitometric analysis and expressed as ratio of MIF/β-actin. * indicates *P *< 0.05 *vs *control cells under normoxia. # *P *< 0.05 *vs *control or scrambled-siRNA transfection under hypoxia. *HIF-1: wild type HIF-1α plasmid transfection; Scrambled-siRNA: scrambled-siRNA plasmid transfection; HIF-1α-siRNA: HIF-1α-siRNA plasmid transfection*.

Silencing HIF-1α expression by HIF-1α-siRNA significantly inhibited hypoxia-induced MIF gene and protein expression in HUASMC, as evaluated by quantitative PCR, Western blot and ELISA (Figure 4). As a negative control, the scrambled-siRNA had no effect on MIF expression in HUASMCs. Furthermore, in order to confirm that HIF-1α specifically induced MIF expression, we utilized a wild type HIF-1α expression vector to over-express HIF-1 during normoxic culture. Over-expression of HIF-1 stimulated MIF expression in HUASMCs under normoxia condition. Thus, we conclude that: 1) HIF-1α is both necessary and sufficient to upregulate MIF expression in HUASMCs, and 2) the hypoxia-induced MIF expression in HUASMCs is most likely mediated by HIF-1α.

### Molecular mechanisms of hypoxia-induced HIF-1α and MIF expression in HUASMCs

Hypoxia activates several intracellular mediators, such as intracellular reactive oxygen species (ROS) [9,20] and extracellular signal-regulated kinase(ERK)[20]. Hypoxia-induced HIF-1α expression and activation was blocked by treatment with antioxidant Tiron (4, 5-dihydroxy-1,3-benzene disulfonic acid, a widely used antioxidant), and PD98059 (inhibitor of ERK signaling)(Figure 2), indicating that activation of ERK signaling and reactive oxygen species generated in response to hypoxia are essential in this process.

**Figure 5:**
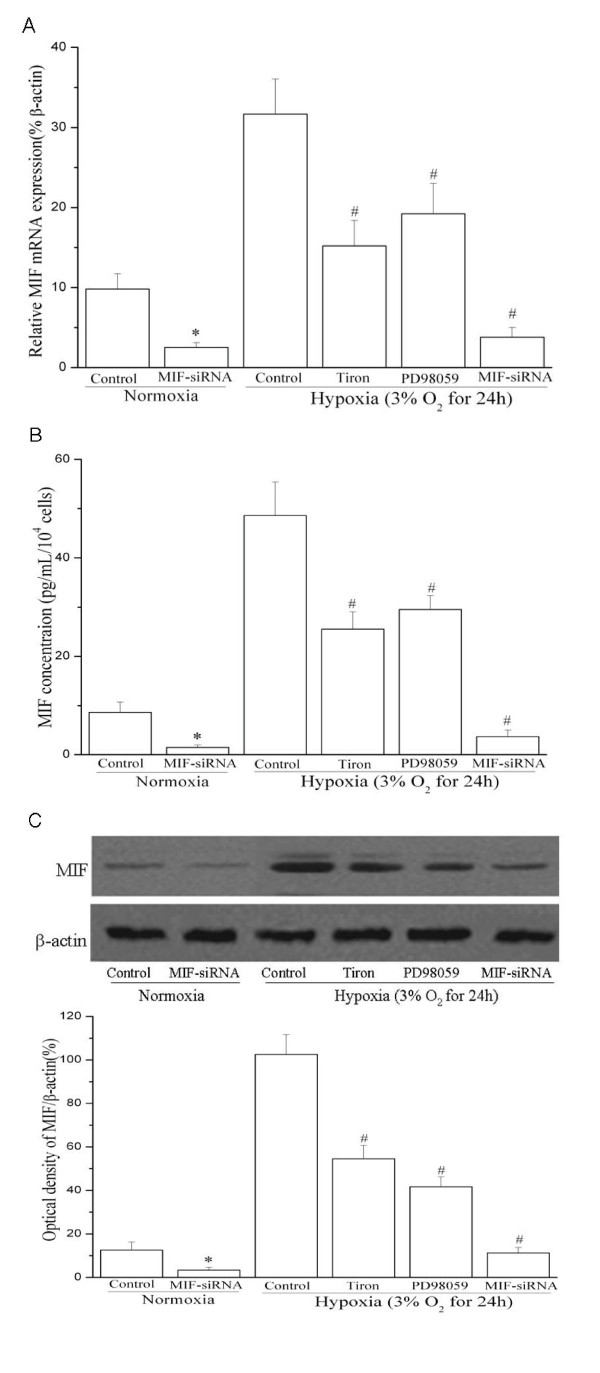
**Molecular mechanisms involved in hypoxia-induced MIF up-regulation**. Cells were pretreated with ERK inhibitor (PD98059, 10^-6 ^mol/L), or anti-oxidant (Tiron, 5 mmol/L) and then exposed to hypoxia (3% O_2_) for 24 hours. In some experiments, cells were transfected with MIF-siRNA for 24 h and then exposed to normoxia or hypoxia (3% O_2_) for 24 h. (A) Real-time PCR results. MIF mRNA expression were assayed by Q-PCR (n = 3 in each group). * indicates *P *< 0.05 *vs *control cells under normoxia. # *P *< 0.05 *vs *control cells under hypoxia. (B) ELISA results. MIF protein released into cell culture media was measured by ELISA (n = 3 in each group). * indicates *P *< 0.05 *vs *control cells under normoxia. # *P *< 0.05 *vs *control cells under hypoxia. (C) Western blot results. Representative Western blot (top) and values of total MIF production (mean ± SEM of 3 experiments, bottom). Results of total MIF protein production were obtained from densitometric analysis and expressed as ratio of MIF/β-actin. * indicates *P *< 0.05 *vs *control cells under normoxia. # *P *< 0.05 *vs *control cells under hypoxia.

Exogenous hydrogen peroxide (H_2_O_2_), a reactive oxygen species (ROS), has been reported to increase MIF expression in normal rat neurons [21]. In our study, we observed that the hypoxia-induced MIF gene expression and protein production was markedly abrogated when the O^2- ^scavenger Tiron was incubated with HUASMCs exposed to hypoxia stimulation. Moreover, inhibition of ERK by its specific inhibitor PD98059 significantly decreased hypoxia-induced MIF expression at both gene and protein levels (Figure 5). These findings suggest that hypoxia-induced MIF expression in HUASMCs is mediated by reactive oxygen species and extracellular signal-regulated kinase signaling.

### Role of MIF in the hypoxia-induced proliferation of HUASMCs

In order to examine if MIF plays a role in hypoxia-induced cell proliferation, we used bromodeoxyuridine (Brdu) incorporation assay[22,23] to study the proliferation of growth-arrested HUASMCs followed by co-treatment with specific MIF-siRNA (Santa Cruz Biotech, USA) and hypoxia (3% O_2_) for 24 h. The specific MIF-siRNA could significantly inhibit MIF expression in HUVSMCs under both normoxia and hypoxia conditions (Figure 5).

**Figure 6:**
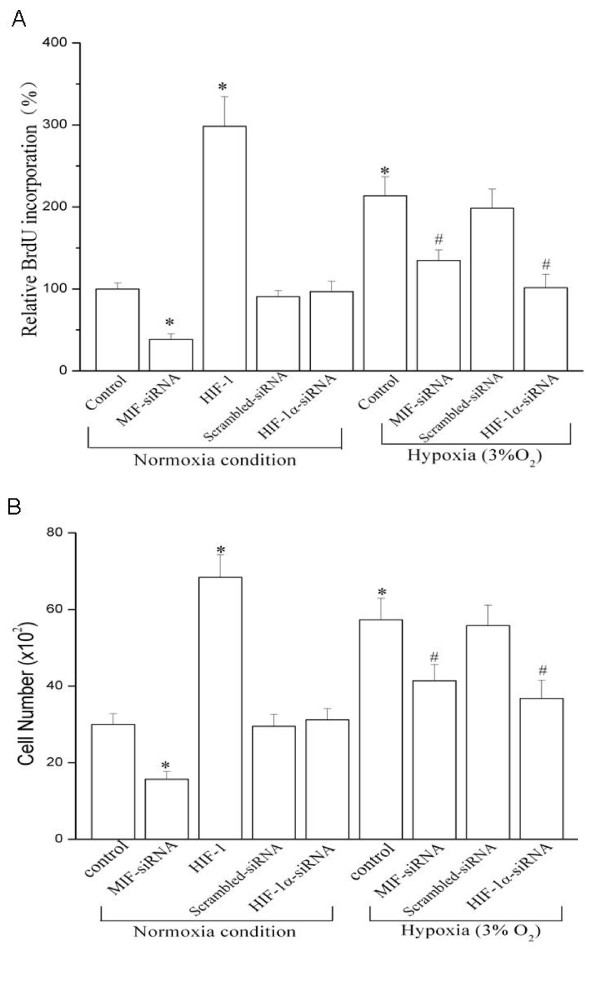
**MIF is involved in hypoxia-induced proliferation and migration of cultured HUASMCs**. Growth-arrested HUASMCs were transfected with wild type HIF-1α, HIF-1α-siRNA, MIF-siRNA or scrambled siRNA for 24 h and then exposed to normoxia or hypoxia (3% O_2_) for 24 h. (A) Cell proliferation measured by BrdU assay (n = 6 in each group). * indicates *P *< 0.05 *vs *control cells under normoxia. # *P *< 0.05 *vs *control or scrambled siRNA transfection under hypoxia. (B) Migration of cultured HUASMCs (n = 3 in each group). * indicates *P *< 0.05 *vs *control cells under normoxia. # *P *< 0.05 *vs *control or scrambled siRNA transfection under hypoxia.

Figure 6A shows that, similar with the other report [17] in which exposure to hypoxia (3% O_2_) stimulated the proliferation of cultured vascular cells, exposure to hypoxia for 24 h increased HUASMCs cell proliferation by 113.6% compared to the cells cultured under normoxia conditions. The siRNA duplex specific to MIF (MIF-siRNA) partly prevented the increase in cell proliferation both under hypoxia (41.5% inhibition) and in normoxia (61.6% inhibition) (Figure 6A). Furthermore, the proliferation of HUASMCs was completely suppressed in HIF-1α-knockdown cells exposed to hypoxia, while it was significantly enhanced in HIF-1α-over expressing cells. Our data indicated that MIF was involved in basal and hypoxia-induced HUASMC proliferation

### Role of MIF in hypoxia-induced migration in HUVSMCs

To examine the effect of MIF in VSMC migration in response to hypoxia, HUASMCs were incubated in a Boyden chamber under normoxic or hypoxic conditions, in the absence or presence of specific siRNA to MIF (MIF-siRNA). As shown in Figure 6B, HUASMCs migrated significantly through the filter membrane under hypoxia (3% O_2_) for 24 h. The MIF-siRNA partly prevented this increase in cell migration both under hypoxia (27.7% inhibition) and in normoxia (47.7% inhibition). Over-expression of HIF-1α could enhance the HUASMC migration under normoxia, whereas knockdown of HIF-1α expression in HUASMCs abolished hypoxia-induced migration in these cells. Our data demonstrate that MIF not only involves in controlling the baseline migration in normoxia condition, but also mediates migration of VSMC in response to hypoxia.

## Discussion

In the present study, the potential correlation between hypoxia and MIF expression was investigated in cultured HUASMCs. The major findings of this study are: (1) hypoxia at 3% O_2_, but not 5 or 10% oxygen, up-regulates MIF expression in VSMCs; (2) transcription factor HIF-1α is necessary and sufficient in the signaling pathway of MIF induction under hypoxia; (3) enhanced generation of reactive oxygen species (ROS) and activation of extracellular signal-regulated kinase (ERK) might be important in mediating the hypoxia-induced expression of HIF-1α and MIF; (4) blockade of MIF results in the inhibition of hypoxia-induced proliferation and migration in the HUASMC cells. Our data clearly indicate that moderate hypoxia plays a crucial role in the modulation of MIF expression in VSMCs. These observations also establish a role of MIF in mediating hypoxia-induced proliferation and migration in vascular smooth muscle cells. In addition, our results suggest that inhibition of MIF might be useful for preventing abnormal VSMC proliferation and migration evoked by hypoxia.

Both MIF and hypoxia play critical roles in inflammation and atherosclerosis. MIF has been identified as a hypoxia-induced gene in cancer cells. Recent evidences show that MIF is up-regulated by hypoxia (1% O_2_) in human cancer cells, such as colon tumor cells [24]and breast carcinoma cells [15]. Moreover, MIF mRNA expression is induced in a HIF-dependent manner in HeLa cells [16]. However, by using siRNA approach, Larsen *et al *[15] reported that MIF regulation in MCF-7 breast cancer cells is due to other hypoxia-induced regulatory mechanisms, independent of HIF-1 and HIF-2 activation. Consistent with previous report [9], we observed that HIF-1α mRNA and protein expression and DNA binding activity could be induced in HUASMCs exposed to hypoxia stimulation (3% O_2_). In our experimental model, over-expression of HIF-1α, the key transcription factor activated during hypoxia, was able to up-regulate MIF gene and protein expression under normoxia, whereas knockdown of HIF-1α expression in HUASMCs inhibited hypoxia-induced MIF expression. Our data demonstrate that, in cultured HUASMCs, hypoxia (3% O_2_) increased MIF expression and synthesis via the HIF-1α pathway, suggesting that the mechanism involved in up-regulation of MIF expression is dependent on cell type.

HIF-1 regulation by non-hypoxic stimuli has gained considerable interest [8,9]. In addition to angiotensin II and thrombin [8], studies have showed that HIF-1α stabilization and activation is also induced by several pro-inflammatory cytokines, such as IL-1 and TNFα [25,26]. As a regulator of MIF expression, HIF-1α might be of potential importance during inflammatory diseases. It is reported that MIF regulates HIF-1 activity in a p53-dependent manner in human cancer cells[27]. Therefore, whether MIF could activate HIF-1α transcriptional activity in VSMCs needs further studies.

Activation of several signaling pathways such as ERK might mediate HIF-1α activation [20] and MIF expression [28]. By using a specific inhibitor, the present observations demonstrate that hypoxia upregulates MIF via activation of ERK in HUVSMCs. Another pathway implicated in the hypoxia induced response is the generation of intercellular ROS [9,20]. Our results showed that the antioxidant Tiron, a cell permeable scavenger of ROS, partially blocks MIF production elicited by hypoxia, suggesting that ROS acts as intermediates of hypoxia-induced MIF expression.

VSMCs proliferation and migration that respond to vascular injury contribute to vessel narrowing and play an important role in the atherosclerotic process. It has been recognized that hypoxia is a stimulus to VSMCs proliferation and migration, a process known as the vascular remodeling [29,30]. Hypoxia plays an important role in vascular remodeling and directly affects VSMCs functions. Some growth factors, such as PDGF [31] and VEGF [29], are involved in the hypoxia-induced proliferation of VSMCs. Consistent with previous reports [23,31], we observed that HIF-1α is essential to VSMC proliferation exposed to hypoxia. Moreover, our results show that the specific MIF-siRNA partially blocks HUASMCs proliferation elicited by hypoxia, suggesting that MIF acts as one of the mediators of hypoxia-induced VSMC proliferation.

In addition to proliferation of VSMCs within the vessel wall, the migration of VSMCs from the media into the neointima is another important feature in the pathogenesis of atherosclerosis [5,32,33]. This process is regulated by multiple factors, and MIF is one of the multiple factors that could increase VSMCs migration[33]. MIF is an important mediator of vessel wall remodeling and acts on the migration of VSMCs in an autocrine and paracrine manner[33]. Previous report shows that hypoxia induces the migration of human coronary artery smooth muscle cells, and the migration is elicited by thrombospondin-1[34]. In the present study, treatment of HUASMCs with the specific MIF-siRNA abolished hypoxia-induced migration, suggesting a role for MIF in the migration of VSMCs in response to hypoxia. Altogether, our data demonstrate that HIF-1α and MIF are both important mediators of vascular cell proliferation and migration.

## Conclusions

In summary, we showed that hypoxia, and specifically HIF-1α, might be a potent and rapid inducer of MIF expression in human VSMCs. The specific MIF-siRNA could suppress both basal and hypoxia-induced proliferation and migration of VSMCs. In view of the important role of MIF and hypoxia to atherosclerosis, our findings might contribute to the understanding of the pathogenesis of progressive atherosclerosis.

## Methods

### Vascular smooth muscle cell culture

Primary cultures of HUASMCs were isolated from freshly delivered umbilical cords by tissue explanting method [35,36] and maintained in DMEM medium (GIBCO-BRL) supplemented with 20% fetal bovine serum (FBS) (Hyclone), 2 mmol/L L-glutamine, and 1% penicillin-streptomycin. All cell cultures were maintained in a humidified 5% CO_2_/95% air incubator at 37°C. When confluent, HUASMCs were passaged every 6-7 days after trypsinization and were used for experiment from the third to sixth passages. HUASMCs were identified by the specific marker of vascular smooth muscle cell (α-smooth muscle actin, α-SMA) immunofluorescence.

The study was conducted according to the Declaration of Helsinki. The study was reviewed and approved by the medical ethics committee of the West China Hospital, Sichuan University.

### Hypoxia stimulation

A humidified temperature controlled incubator model IG 750 (Jouan, Saint-Nazaire, France) was used as a hypoxic chamber. For hypoxia conditions, the concentration of oxygen was reduced to 10, 5, or 3% by replacement with N_2_, and CO_2 _was constant at 5%. Control was defined as 95% air and 5% CO2.

HUASMCs were seeded in the 60 mm plates (Corning, NY, USA). All cells were grown to 90% confluence under normoxic conditions. The media was then changed, pretreated in starving conditions (normal culture media without FBS) for 24 h before exposure to hypoxia. Experiments were carried out with three replicates for each data point. In some experiments, cells were pre-treated for 30 minutes with 10^-6 ^mol/L PD98059, or 5×10^-3 ^mol/L Tiron, and then exposed to hypoxia for 24 h.

### RNA interference

HIF-1α target-specific siRNA plasmid vector and a scrambled siRNA control plasmid were constructed as our previously described [19]. Constructs expressing HIF-1α (wild type) were a gift from Professor Cormac T. Taylor (University College, Dublin, Ireland). Transient transfections were performed using the cationic lipid, Lipofectamine™ 2000 (Invitrogen, USA), according to the manufacturer's protocols. Cells were plated in dishes (60 mm in diameter) grown to 90% confluence prior to transfection. Each dish was transfected with 8.0 μg of plasmid containing HIF siRNA or control RNA (plasmids containing null mutant HIF-GFP) and 20 μL of Lipofectamine™ 2000. Wild type HIF-1α was co-transfected with EGFP. Transfection efficiency averaged between 60-70% as measured by green fluorescent protein expression. Cells were allowed to recover in DMEM for 6 h after transfection. After the medium changed, the culture was exposed to normoxia or hypoxia for 24 h before assays were performed.

In some experiments, cultured HUASMCs were transfected with 800 ng siRNA duplex specific to MIF (MIF-siRNA) (Santa Cruz Biotech, USA) according to the manufacturer's instruction.

### Real-time RT-PCR

The expression of MIF gene was identified by quantitative real time RT-PCR (Q-PCR) as reported earlier [37]. Total RNA was extracted from HUASMCs cells using TRIZOL^® ^reagent (Invitrogen, USA). Q-PCR was carried out on an ABI Prism 7300 PCR Detection System (Applied Biosystems, USA) with fluorescence dye SYBR Green (SYBR Green Real-time PCR Master Mix, TOYOBO, Japan). The sequences of the primers were as follows: MIF-F: 5'-GTGCCAGAGGGGTTTCTCTC-3', MIF-R: 5'-CAGCAGCTTGCTGTAGTTGC-3'. HIF-1α-F: 5'-CTAGCCGAGGAA GAACTATGAACAT-3', HIF-1α-R: 5'-CTGAGGTTGGTTACTGTT GGTATCA-3'. β-actin-F: 5'-CAACTCCATCATGAAGTGTAAC-3'; β-actin-R: 5'-CCACACGGA GTACTTGCGCTG-3'. The thermal cycling conditions were as following: 95°C 60 seconds, 40 cycles of 95°C for 15 seconds, 58°C for 15 seconds, 72°C for 45 seconds (data collection). Data analysis was carried out by ABI sequence detection software using relative quantification. For quantification, the target sequence was normalized to the β-actin mRNA levels.

### Western Blot Analysis

Western-blot analysis of MIF and HIF-1α was performed using rabbit polyclonal antibodies against MIF or HIF-1α (Santa Cruz Biotechnology, Inc., Santa Cruz, CA, USA), according to the method described before [38,39]. In brief, HUASMC cells were scraped from dishes and cellular protein extracts were prepared by homogenization in an ice-cold RIPA lysis buffer containing protease inhibitor cocktail (Roche, Indianapolis, IN, USA). Cell lysates (40 μg) were separated by denaturing 10% SDS-PAGE and then transferred to polyvinylidene difluoride (PVDF) membrane (Millipore) using a MiniProtein III system (Bio-Rad, CA, USA). Transferred proteins were probed with the rabbit polyclonal anti-MIF or anti-HIF-1α antibody (1:250) and visualized using the horseradish peroxidase conjugated secondary anti-rabbit (1:3000; Santa Cruz Biotechnology, Inc) antibody and ECL solution (Pierce, USA). Equal protein loading was verified by reprobing the membrane with an anti β-actin antibody (Santa Cruz Biotechnology, Inc.). For quantification purposes, densitometry measurements were performed using Quantity One^® ^image analysis software for Windows (Bio-Rad). All specific blot values were corrected for β-actin expression.

### Quantification of MIF by ELISA

The level of MIF in culture supernatants was measured by ELISA using ELISA kit (Quantikine ELISA, R&D Systems, USA) according to manufacturer's instructions [15,38]. In each experiment the MIF concentrations was normalized to cell count.

### Preparation of Nuclear Extracts and Electrophoretic Mobility Shift Assay

Nuclear extracts from HUASMCs were prepared according to standard protocols [18]. Electrophoretic mobility shift assay (EMSA) was performed as described [18], using a 24-bp oligonucleotide probe containing the HRE with the HIF-1α binding site and adjacent flanking regions of human MIF gene [18]. The probe was as follows: 5'-TCTGT***ACGTG***ACCACACTCACCTC-3' (The consensus core HIF-1α binding sites are boldfaced).

### Assessment of cell proliferation

The effect of hypoxia on cell proliferation was determined by the bromodeoxyuridine (BrdU) incorporation assay, as described previously [22,23]. Briefly, HUASMC cells were subcultured in 96-well plates and incubated with serum-free medium for 24 h in order to synchronize the cells in G_0_/G_1 _phase. Quiescent cells were transfected with wild type HIF-1α, HIF-1α-shRNA, MIF-shRNA or scrambled siRNA for 24 hours, and then either exposed to normoxia or hypoxia for 24 h. 10 μL of BrdU (100 μM) was added to each well and incubated for the last 3 h of treatment. BrdU incorporation was measured by a colorimetric ELISA kit (Roche, Indianapolis, IN, USA). The light absorbance at 450 nm was detected using ELISA reader (Bio-Rad Model 680, USA).

### Cell Migration Assay

The migration of HUASMCs was determined by using a Matrigel chamber system (BD Biosciences) as previous reports [34,40]. Briefly, HUASMC cells were incubated with serum-free medium for 24 h. Quiescent cultures were transfected with wild type HIF-1α, HIF-1α-siRNA, MIF-siRNA or scrambled siRNA for 24 h. Following transfection, HUASMCs (1×10^4^) were suspended in 0.5 ml of culture medium and added to the upper chamber. The upper chamber was lodged into the lower chamber containing 0.75 ml of culture medium. After incubating at 37°C for 24 h under normoxia or hypoxia conditions, the cells in the upper side of the filter membrane were removed with cotton swabs. Then the cells in the lower side were trypsinized, and the number of cells in the lower side was counted.

### Statistical analysis

The experimental data were expressed as means ± SD. Group means were compared by ANOVA using the statistical software program SPSS 10.0 for Windows (Chicago, IL, USA), and *P *value < 0.05 was considered statistically significant in all cases.

## Authors' contributions

HF conceived of the experiments, carried out all experiments and prepared the manuscript. FL and LY conceived of the experiments. WW performed cell culture. XL provided expert advice and interpretation of the study's results. All authors read and approved the final manuscript.
